# Comparative Evaluation of the Remineralizing Potential of Flaxseed Paste, Chicken Eggshell Paste, and Fluoride Toothpaste on the Enamel of Primary Teeth Using Scanning Electron Microscopy-Energy Dispersive X-ray Analysis: An In-Vitro Study

**DOI:** 10.7759/cureus.60040

**Published:** 2024-05-10

**Authors:** Farah Shehani A, Kavitha Ramar

**Affiliations:** 1 Pediatric and Preventive Dentistry, SRM Institute of Science and Technology, Chennai, IND

**Keywords:** sem edx, new trends in pediatric dentistry, restorative dentistry, primary molar teeth, casein-phosphopeptide-amorphous calcium phosphate, calcium release, fluoride releasing, tooth remineralization, egg shell powder, flax seeds

## Abstract

Introduction: Dental caries in primary teeth remains a critical public health challenge globally. Although fluoride toothpaste is the standard care for remineralization, its efficacy is limited by the requirement for bioavailable calcium and phosphate ions and its diminished performance on irregular dental surfaces. This study evaluates natural alternatives for dental care, focusing on their mineralizing potential compared to fluoride.

Aim: This study aims to assess and compare the efficacy of remineralization by flaxseed paste and chicken eggshell paste to that of standard fluoride toothpaste on artificially demineralized primary teeth.

Materials and Methods: We utilized an in vitro model, creating standardized white spot lesions on extracted primary teeth to simulate early carious lesions. The teeth were treated with preparations of flaxseed paste, chicken eggshell paste, and fluoride toothpaste. Remineralization was quantitatively analyzed using scanning electron microscopy-energy dispersive x-ray analysis (SEM-EDX) conducted with a high-resolution scanning electron microscope (HRSEM) from Thermoscientific Apreo S at Sir C V Raman Research Park, SRM Institute of Science & Technology, Kattankulathur, Chennai, Tamil Nadu.

Results: Quantitative analysis revealed that both flaxseed and chicken eggshell pastes not only met but, in some cases, exceeded the remineralization performance of fluoride toothpaste. Significant differences were observed in the deposition of calcium and phosphate ions on the lesion surfaces.

Conclusion: The study conducted at the Department of Pediatric and Preventive Dentistry at SRM Dental College, Kattankulathur, confirms the potential of flaxseed and chicken eggshell pastes as viable, cost-effective, and accessible alternatives to fluoride toothpaste for the remineralization of enamel in primary teeth. These findings support the inclusion of these natural agents in oral hygiene regimens and underscore the importance of further research into holistic approaches for the prevention and treatment of dental caries in children.

## Introduction

Dental caries is a prevalent public health concern affecting millions globally, predominantly driven by a dynamic interplay between demineralization and remineralization processes within the oral cavity. This disease affects various age groups but is particularly problematic in pediatric populations due to unique challenges in managing young patients. The disease progresses through cycles of enamel weakening and strengthening influenced by a combination of pathological factors such as fermentable carbohydrates, cariogenic bacteria, and salivary dysfunctions, and protective factors including antibacterial agents, remineralizing ions, and effective saliva flow. Early indicators of enamel caries, such as white spot lesions (WSL), signal the onset of the disease and can be effectively managed or prevented through the application of ions like fluoride, phosphate, or calcium [1-3].

Historically, fluoride has been revered as the gold standard in caries prevention since the 1950s, renowned for its ability to inhibit demineralization, enhance remineralization, and offer antibacterial properties. However, despite widespread fluoride use, recent epidemiological data indicates a troubling trend: caries rates have stagnated or even increased in certain demographic groups. Moreover, in the wake of holistic dentistry movements and the classification of fluoride as a chemical neurotoxicant, public and scientific communities have raised safety concerns regarding the use of high-concentration fluoride products, particularly in children who are at risk of developing dental fluorosis [4].

Given the narrow “dose gap” between the beneficial effects of caries depletion and the adverse toxic effects of fluoride, public health officials have set limits on fluoride concentrations in over-the-counter toothpaste to between 1,000 and 1,500 ppm. For children under six, this dosage is even lower, underscoring the need for alternative remineralization strategies that can enhance or complement the effects of fluoride. The search for new remineralization agents has led to an increased interest in natural products due to their safety profile, minimal side effects, and effectiveness in dental applications [5-7].

In recent years, research has highlighted organic vegetables and food supplements as promising agents for promoting oral health. Compounds derived from plants such as flaxseed offer an alternative to synthetic chemicals for plaque control and prevention of demineralization. Flaxseed, with its rich content in minerals like calcium, magnesium, phosphorus, and potassium, alongside antibacterial lignans, has shown potential in fighting cariogenic bacteria like Streptococcus mutans, positioning it as a viable natural remedy. Additionally, the medicinal use of eggshell powder as a natural source of calcium, as observed in its application in bone substitute materials and the regeneration of bone defects, suggests its potential in dental remineralization [8-10].

Chicken eggshell powder (CESP), in particular, has been noted for its high calcium carbonate content, and recent studies have explored its use in vitro to enhance bone mineral density and stimulate cartilage growth [11]. Although limited, some research has also begun to evaluate the effectiveness of CESP in remineralizing incipient enamel carious lesions on primary teeth.

The purpose of this study is to assess and compare the remineralization potential of organic flax seed paste, chicken eggshell paste, and traditional fluoride toothpaste on artificially created WSL in primary teeth. This research aims to contribute to the field by exploring effective, natural, and safe alternatives to conventional fluoride treatments, potentially revolutionizing pediatric dental care by leveraging the synergistic effects of natural compounds to enhance oral health outcomes.

## Materials and methods

This laboratory study was conducted in April 2023 at the Department of Pediatric and Preventive Dentistry at SRM Dental College, Kattankulathur. It received approval from the Institutional Review Board and the Ethical Committee under the reference SRMIEC-ST0523-550. Standardized WSL were created on extracted primary teeth to simulate early carious lesions. Treatments with flaxseed paste, chicken eggshell paste, and fluoride toothpaste were applied, and their effects on enamel remineralization were assessed using Scanning Electron Microscopy-Energy Dispersive X-ray Analysis (SEM-EDX) with a High-Resolution Scanning Electron Microscope (HRSEM) manufactured by Thermosceintific Apreo S at Sir C V Raman Research Park, SRM Institute of Science & Technology, Kattankulathur, Chennai, Tamil Nadu. Specific parameters were set for the mineral content analysis, including an acceleration voltage of 20 kV, a working distance of 10 mm, and a spot size of 5.5. EDX scans were performed with a take-off angle of 35 degrees, optimal for elemental analysis, and dwell times of 100 microseconds per pixel were used to ensure adequate signal acquisition without damaging the sample.

Aim and objective of the study

The primary aim of this study was to evaluate and compare the remineralization efficacy of flaxseed paste and chicken eggshell paste with that of standard fluoride toothpaste on artificially demineralized primary teeth. The objective was to investigate whether these natural substances could serve as cost-effective, accessible alternatives to fluoride toothpaste, potentially enhancing enamel remineralization in primary teeth.

Type of study

This was a controlled laboratory study designed to simulate early carious lesions in primary teeth and assess the remineralization potential of different treatments. The experimental setup allowed for precise quantification and comparison of the effects of natural pastes and fluoride toothpaste on tooth enamel.

Criteria for selection

**Figure 1:**
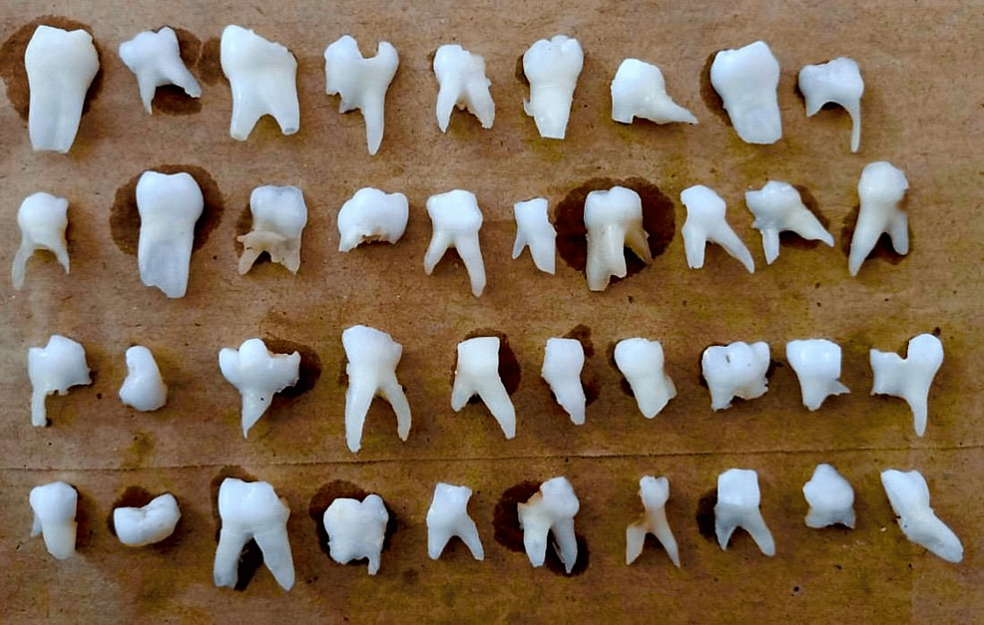
Twenty non-decayed upper and lower primary molars with intact crowns were longitudinally bisected, yielding a total of forty samples.

Inclusion criteria include selected were non-decayed upper and lower primary molars with crowns intact (Figure 1).

Exclusion criteria include any teeth with signs of restorations, decay, surface impairments, caries, under-calcification, fluorosis, cracks in the enamel, previous chemical interventions, as well as any adult teeth, were excluded from the study.

Estimation of sample size

The study determined a need for 40 samples based on calculations using G power software targeting a 95% power and a 5% margin of error, leading to the inclusion of 20 upper and lower primary teeth.

Procedural methodology

The selected non-decayed primary molars were initially cleaned using an ultrasonic scaler to remove any adherent debris and calculus without damaging the enamel. Following this, the teeth were further cleaned with pumice and a rubber cup to ensure a completely clean surface, which is essential for the accuracy of our subsequent remineralization assessments. The teeth were then bisected longitudinally using a high-precision diamond disc cutter. The specific model used for this procedure was the IsoMet Low-Speed NMD dental double-Sided diamond discs with mandrel, manufactured by Nexus Medodent, Mulund West, Mumbai, Maharashtra 400080. This device is equipped with a diamond wafering blade designed specifically for delicate sectioning of hard materials, ensuring minimal sample deformation and loss.

Storage Conditions

The bisected teeth samples were stored in distilled water to prevent dehydration and to maintain the physiological state of the dental tissues. The storage was carried out at room temperature, which was consistently maintained at 23°C (±1°C). This temperature was chosen to mimic ambient environmental conditions, thus avoiding any thermal stress that could potentially alter the physical properties of the tooth enamel and dentin.

Duration of Storage

**Figure 2:**
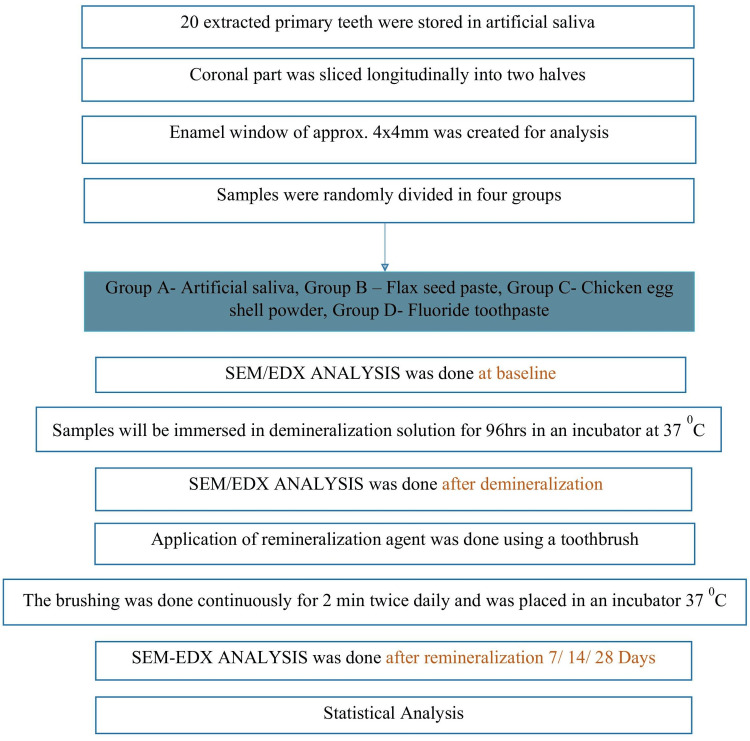
Consort diagram depicting the study protocol.

The samples were stored for a period of 48 hours before the commencement of the remineralization treatments. This duration was determined based on preliminary studies indicating that this time frame is sufficient to stabilize the tooth samples post-sectioning without inducing significant changes in the hydroxyapatite structure of the enamel, following the protocol outlined in the CONSORT diagram (Figure 2).

Formation of artificial WSLs

A demineralizing mixture was prepared by combining 0.4723g calcium nitrate, 0.2722g potassium dihydrogen phosphate, and 4.5083g acetic acid in a liter of distilled water, adjusting the solution's pH to between 4 and 4.5 using 50% NaOH [12]. Except for a designated 4x4mm region, the buccal surfaces of the teeth were covered with a clear, acid-resistant varnish. These 40 specimens were then fully submerged in the solution and placed in an incubator at 37°C for 96 hours. Following this period, the emergence of WSLs was verified both visually and via SEM/EDX analysis using a HRSEM (manufactured by Thermoscientific Apreo S).

The incubator used was the ORBITEK model of SCIGENICS BIOTECH, with precise temperature control set to maintain a constant 37°C and equipped with active humidity control to prevent evaporation of the solution, ensuring consistent experimental conditions. After a period of 96 hours within this controlled environment, the formation of WSLs was confirmed visually. The visual verification of WSLs was conducted under standardized lighting conditions using a dental examination light and a dental loupe with 3.5x magnification, where each tooth was dried for 10 seconds to enhance visibility of demineralization, appearing as opaque white areas. Each sample was independently evaluated by two experienced dental researchers, with discrepancies resolved through consensus. Following visual confirmation, SEM/EDX analysis was performed using a HRSEM manufactured by Thermo Scientific Apreo S. Analysis parameters included magnification levels from 500x to 10,000x, utilizing secondary electron imaging for topographical evaluation and backscattered electron imaging for compositional contrasts. The EDX settings operated in point and scan mode for localized chemical analysis, particularly quantifying calcium and phosphate changes, using Thermo Scientific™ Pathfinder X-ray Microanalysis Software for detailed elemental mapping and quantification. These expanded methodologies ensure a thorough assessment and robust foundation for our conclusions on the tested remineralization agents.

Post-WSL development, the teeth were categorized into four experimental groups:

(1) A control group was treated with artificial saliva (N=10).

(2) A group treated with a paste made from flaxseed (N=10).

(3) A group that received chicken eggshell paste (N=10).

(4) A group that received fluoride toothpaste application (N=10).

Preparation of synthetic saliva

The formulation of the synthetic saliva was based on standard compositions referenced in the literature to mimic the ionic and buffering properties of natural saliva. Specifically, our concoction followed the guidelines outlined by Kleinberg et al., which are widely accepted for studies requiring the simulation of oral cavity conditions. A concoction consisting of 0.75g sodium azide, 0.804g potassium mono hydrogen phosphate, 0.166g calcium chloride, 0.59g magnesium chloride, and 1.02g sodium chloride was mixed into one liter of distilled water to achieve a target pH of around 7. The target pH of approximately 7.0 was precisely achieved by initially measuring the pH after the initial mixing of the components using a calibrated digital pH meter. Minor adjustments were then made by adding minute quantities of sodium hydroxide or hydrochloric acid until the pH stabilized at 7.0 ± 0.05. This synthetic saliva was used to immerse the dental samples in individual containers at a temperature of 37°C, maintained via a thermostatically controlled water bath. This setup was chosen to closely simulate the thermal and chemical conditions within the human oral cavity to mimic the conditions within the oral cavity.

Process of remineralization

The baseline group underwent no specific treatment. A paste made from ground flaxseed (OKRAA, India) (DCGI 42)/ (FSSAI 10020021005537), mixed with deionized distilled water at a ratio of 1g/mL was used for one of the treatment groups. Similarly, a solution consisting of CESP (Pure only, India) (DCGI 42)/(EPM101R1S), dissolved in sterile deionized water to the same concentration was prepared for another group. A commercial fluoridated toothpaste (Colgate Total 12, India), with 0.22% Sodium Fluoride equating to w/w 1,500 ppm was utilized for the final treatment group. Each specimen was brushed using the allocated treatment without being rinsed off, this procedure was performed twice a day for three minutes and followed by incubation at 37°C in newly prepared artificial saliva each day.

Specimen evaluation

By the 29th day, a pair of samples from each treatment category was thoroughly cleaned, dehydrated using 100% ethanol, and left to air dry. The cleaned and dehydrated samples were further affixed to SEM stubs using conductive carbon adhesive and subsequently coated with a 20 nm layer of gold, applied via a sputtering device (Quorum, Q150TS Plus) under an argon atmosphere to ensure even deposition. To mitigate the introduction of artifacts, several measures were implemented: the sputtering duration was meticulously controlled to achieve uniform coating thickness, samples underwent a systematic dehydration process using progressively concentrated ethanol solutions to prevent tissue distortion, air drying was conducted under controlled environmental conditions to avert thermal stresses, and a reduced acceleration voltage of 20 kV was utilized during SEM imaging to minimize beam damage and charging effects. These protocols were essential to preserve the integrity of the surface and mineral composition analysis, ensuring that the SEM images represented the actual condition of the samples following treatment.

Safety protocols

During the experiment, comprehensive safety protocols were followed to ensure the safe handling and disposal of materials used, particularly when working with hazardous chemicals such as sodium azide and acetic acid. All personnel were required to wear appropriate personal protective equipment (PPE), including gloves, goggles, and lab coats. Chemicals were handled in well-ventilated areas, and all waste was disposed of according to institutional safety guidelines and environmental regulations to minimize any risk of exposure or environmental contamination.

Statistical examination

Paired t-tests were conducted to analyze the changes in mineral content detected through SEM-EDX. These tests were two-tailed, considering the potential for both increases and decreases in mineral content, with a significance level set at p<0.05. Assumptions of normality and homogeneity of variances were verified using the Shapiro-Wilk test and Levene's Test, respectively. Additionally, ANOVA was applied to evaluate differences across treatment groups, followed by post-hoc Tukey tests to identify specific group differences in calcium-to-phosphorus ratios.

## Results

Employing a one-way ANOVA test to assess the calcium/phosphorus mass percentages after the remineralization process, the data highlighted CESP peaking at 35.84 ± 0.79% calcium on day 28, the highest value noted during the study. In contrast, the flaxseed treatment showed the highest phosphorus percentage on the same day, with these differences being statistically significant, denoted by a p-value under 0.001, as outlined in Tables 1, 2.

**Table 1:** Intergroup comparison of Calcium Weight% between various remineralizing agents and control group. *Statistically Significant (p<0.05); F value – One way ANOVA; Wt% of Ca2+ - weight percentage of calcium ions; Mean ± SD- mean ± standard deviation; Groups- Various remineralizing agents (flax seed paste, chicken eggshell powder, fluoride toothpaste, and artificial saliva).

Wt% of Ca^2+^	Groups	Mean ± SD	F-value	P-value
Baseline	Flax seed paste	11.13 ± 2.07	2.102	0.117
Baseline	Chicken eggshell powder	12.21 ± 1.78	2.103	0.118
Baseline	Fluoride toothpaste	10.06 ± 1.68	2.104	0.119
Baseline	Artificial Saliva	11.13 ± 2.07	2.105	0.120
Day 7	Flax seed paste	25.12 ± 1.48	1314.210	0.000*
Day 7	Chicken eggshell powder	28.64 ± 0.95	1314.211	0.000*
Day 7	Fluoride toothpaste	27.38 ± 1.58	1314.212	0.000*
Day 7	Artificial Saliva	0.00 ± 0.00	1314.213	0.000*
Day 14	Flax seed paste	31.62 ± 2.68	1019.065	0.000*
Day 14	Chicken eggshell powder	31.34 ± 1.25	1019.066	0.000*
Day 14	Fluoride toothpaste	31.74 ± 1.00	1019.067	0.000*
Day 14	Artificial Saliva	0.00 ± 0.00	1019.068	0.000*
Day 28	Flax seed paste	31.32 ± 1.54	2466.793	0.000*
Day 28	Chicken eggshell powder	35.84 ± 0.79	2466.794	0.000*
Day 28	Fluoride toothpaste	34.83 ± 1.31	2466.795	0.000*
Day 28	Artificial Saliva	0.00 ± 0.00	2466.796	0.000*

**Table 2:** Intergroup comparison of Phosphorous Weight% between various remineralizing agents and control group. *Statistically Significant (p<0.05); F value – One way ANOVA; Wt% of P -weight percentage of phosphorus, Mean±SD- mean ± standard deviation; Groups- Various remineralizing agents (flax seed paste, chicken eggshell powder, fluoride toothpaste, and artificial saliva).

Wt% of P	Groups	Mean±SD	F-value	P-value
Baseline	Flax seed paste	8.00±1.43	0.541	0.657
Baseline	Chicken eggshell powder	8.40±0.83	0.542	0.658
Baseline	Fluoride toothpaste	7.60±1.75	0.543	0.659
Baseline	Artificial Saliva	8.00±1.43	0.544	0.660
Day 7	Flax seed paste	8.00±0.97	0.080	0.970
Day 7	Chicken eggshell powder	8.19±1.12	0.081	0.971
Day 7	Fluoride toothpaste	8.05±0.95	0.082	0.972
Day 7	Artificial Saliva	8.00±0.97	0.083	0.973
Day 14	Flax seed paste	12.30±1.78	17.588	0.000*
Day 14	Chicken eggshell powder	12.30±1.78	17.589	0.000*
Day 14	Fluoride toothpaste	12.81±2.04	17.590	0.000*
Day 14	Artificial Saliva	8.00±0.97	17.591	0.000*
Day 28	Flax seed paste	14.40±0.94	51.613	0.000*
Day 28	Chicken eggshell powder	14.30±0.99	51.614	0.000*
Day 28	Fluoride toothpaste	12.81±2.04	51.615	0.000*
Day 28	Artificial Saliva	8.00±0.97	51.616	0.000*

**Figure 3:**
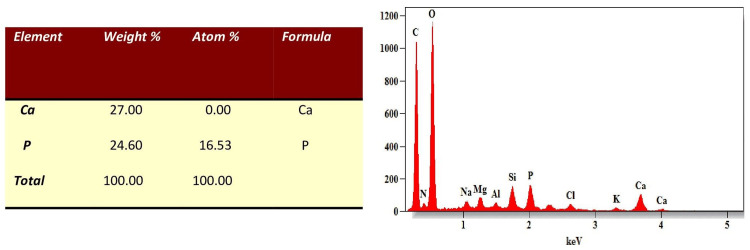
EDX evaluations of Ca/P mass percentage before demineralization. Ca: Calcium, P: Phosphorus, weight%- weight percentage, atom%- atomic weight percentage, keV-kiloelectronvolt, EDX - Energy Dispersive X-ray.

**Figure 4:**
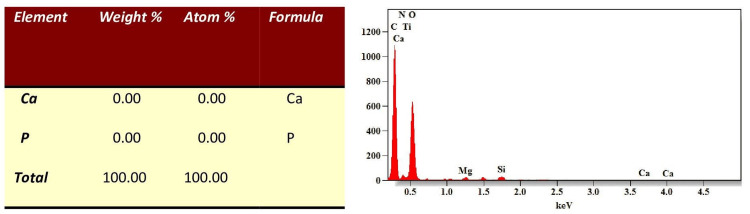
EDX evaluations of Ca/P mass percentage after demineralization. Ca: Calcium, P: Phosphorus, weight%- weight percentage, atom%-atomic weight percentage, keV-kiloelectronvolt, EDX - Energy Dispersive X-ray.

EDX evaluations provided quantitative insights into the Ca/P ratios at various points: initial assessment, post-demineralization, and after 7, 14, and 28 days of remineralization. A significant decrease in the Ca/P mass percentage was observed after demineralization when compared to the initial levels as seen in Figures 3, 4.

Subsequent post-hoc analysis at specific intervals revealed marked improvements in the mean Ca/P mass percentages from after demineralization to after remineralization for the specimens treated with CESP, Flaxseed Paste, and Fluoride Toothpaste, particularly noticeable at the 14 and 28-day intervals. These enhancements were statistically profound, with a p-value below 0.001, as shown in Tables 3, 4. The CESP and Flaxseed Paste treatments showed especially significant increases in Ca/P mass percentage shortly after the remineralization began, notably on days 7 and 14.

Comparisons within groups, conducted through the post-hoc Tukey test after both demineralization and remineralization stages, demonstrated that the Chicken Eggshell and Flaxseed treatments significantly elevated the average Ca/P mass percentages, affirming the statistical relevance of these findings for all groups involved, as depicted in Tables 3, 4.

**Table 3:** Intragroup comparison for the comparison of the Weight% of Calcium between various time periods. *Mean difference is significant at (p<0.05)- Test used: Post hoc Tukey test. The data is represented in: I (time periods), J (time periods), Mean difference (I-J), Dependent variable: Calcium wt% - calcium weight percentage in Flax Seeds paste, chicken eggshell powder, fluoride toothpaste, and artificial saliva).

Dependent Variable	(I) Time Periods	(J) Time Periods	Mean Difference (I-J)	P-value
Calcium wt% in Flax Seeds	Baseline	7 day	-13.99200^*^	0
Calcium wt% in Flax Seeds	Baseline	14 day	-20.49200^*^	0
Calcium wt% in Flax Seeds	Baseline	28 day	-20.19200^*^	0
Calcium wt% in Flax Seeds	7 day	14 day	-6.50000^*^	0
Calcium wt% in Flax Seeds	7 day	28 day	-6.20000^*^	0
Calcium wt% in Flax Seeds	14 day	28 day	0.3	0.987
Calcium wt% in Chicken eggshell powder	Baseline	7 day	-16.43000^*^	0
Calcium wt% in Chicken eggshell powder	Baseline	14 day	-19.13000^*^	0
Calcium wt% in Chicken eggshell powder	Baseline	28 day	-23.63000^*^	0
Calcium wt% in Chicken eggshell powder	7 day	14 day	-2.70000^*^	0
Calcium wt% in Chicken eggshell powder	7 day	28 day	-7.20000^*^	0
Calcium wt% in Chicken eggshell powder	14 day	28 day	-4.50000^*^	0
Calcium wt% in Fluoride toothpaste	Baseline	7 day	-17.32100^*^	0
Calcium wt% in Fluoride toothpaste	Baseline	14 day	-21.68200^*^	0
Calcium wt% in Fluoride toothpaste	Baseline	28 day	-24.77000^*^	0
Calcium wt% in Fluoride toothpaste	7 day	14 day	-4.36100^*^	0
Calcium wt% in Fluoride toothpaste	7 day	28 day	-7.44900^*^	0
Calcium wt% in Fluoride toothpaste	14 day	28 day	-3.08800^*^	0
Calcium wt% in Artificial Saliva	Baseline	7 day	11.13700^*^	0
Calcium wt% in Artificial Saliva	Baseline	14 day	11.13700^*^	0
Calcium wt% in Artificial Saliva	Baseline	28 day	11.13700^*^	0
Calcium wt% in Artificial Saliva	7 day	14 day	0	1
Calcium wt% in Artificial Saliva	7 day	28 day	0	1
Calcium wt% in Artificial Saliva	14 day	28 day	0	1

**Table 4:** Intragroup comparison of the weight% of phosphorus between various time periods. *Mean difference is significant at (p<0.05)- Test used: Post hoc Tukey test. The data is represented in: I (time periods), J (time periods), Mean difference (I-J), and dependent variable: Phosphorous wt%- phosphorous weight percentage in flax seed paste, chicken eggshell powder, fluoride toothpaste, and artificial saliva).

Dependent Variable	(I) Time Periods	(J) Time Periods	Mean Difference (I-J)	P-value
Phosphorous wt% in Flax Seeds	Baseline	7 day	0	1
Phosphorous wt% in Flax Seeds	Baseline	14 day	-4.30000^*^	0
Phosphorous wt% in Flax Seeds	Baseline	28 day	-6.40000^*^	0
Phosphorous wt% in Flax Seeds	7 day	14 day	-4.30000^*^	0
Phosphorous wt% in Flax Seeds	7 day	28 day	-6.40000^*^	0
Phosphorous wt% in Flax Seeds	14 day	28 day	-2.10000^*^	0.006
Phosphorous wt% in Chicken eggshell powder	Baseline	7 day	0.211	0.981
Phosphorous wt% in Chicken eggshell powder	Baseline	14 day	-3.89900^*^	0
Phosphorous wt% in Chicken eggshell powder	Baseline	28 day	-5.90000^*^	0
Phosphorous wt% in Chicken eggshell powder	7 day	14 day	-4.11000^*^	0
Phosphorous wt% in Chicken eggshell powder	7 day	28 day	-6.11100^*^	0
Phosphorous wt% in Chicken eggshell powder	14 day	28 day	-2.00100^*^	0.005
Phosphorous wt% in Fluoride toothpaste	Baseline	7 day	-0.446	0.941
Phosphorous wt% in Fluoride toothpaste	Baseline	14 day	-5.21000^*^	0
Phosphorous wt% in Fluoride toothpaste	Baseline	28 day	-5.21000^*^	0
Phosphorous wt% in Fluoride toothpaste	7 day	14 day	-4.76400^*^	0
Phosphorous wt% in Fluoride toothpaste	7 day	28 day	-4.76400^*^	0
Phosphorous wt% in Fluoride toothpaste	14 day	28 day	0	1
Phosphorous wt% in Artificial Saliva	Baseline	7 day	0	1
Phosphorous wt% in Artificial Saliva	Baseline	14 day	0	1
Phosphorous wt% in Artificial Saliva	Baseline	28 day	0	1
Phosphorous wt% in Artificial Saliva	7 day	14 day	0	1
Phosphorous wt% in Artificial Saliva	7 day	28 day	0	1
Phosphorous wt% in Artificial Saliva	14 day	28 day	0	1

**Figure 5:**
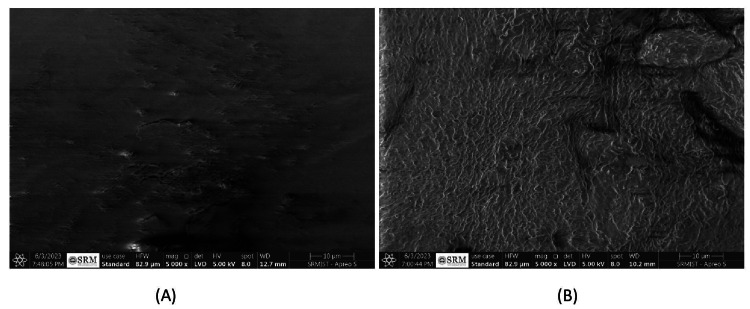
(A) SEM image of enamel before demineralization, (B) SEM image of enamel after demineralization.

Summary of SEM analysis using a HRSEM (manufactured by Thermoscientific Apreo S): Initial observations with a Scanning Electron Microscope (SEM) of intact enamel showcased a smooth, consistent texture, marked by neatly arranged enamel crystals and rods (Figure 5A). In contrast, enamel subjected to artificially induced White Spot Lesions (WSL) demonstrated significant porosity, signaling a loss of enamel integrity and disarray in the crystal structure (Figure 5B).

**Figure 6:**
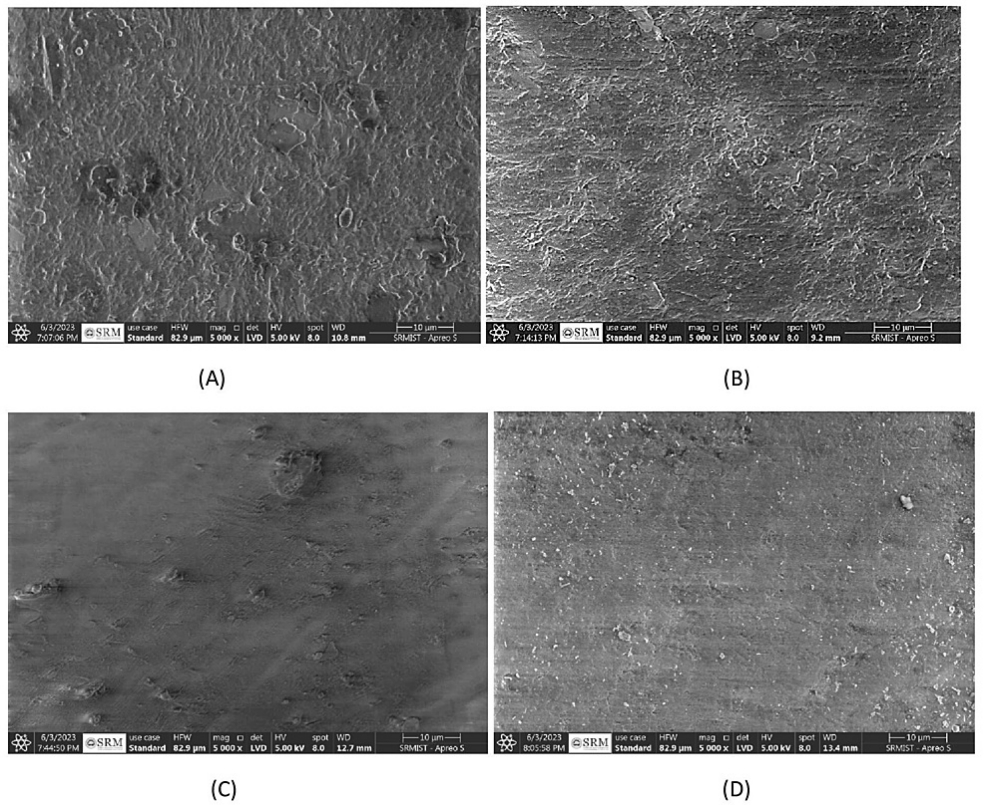
(A) SEM image of remineralized enamel after seven days in GROUP 1, (B) SEM image of remineralized enamel after seven days in GROUP 2, (C) SEM image of remineralized enamel after seven days in GROUP 3, and (D) SEM image of remineralized enamel after seven days in GROUP 4.

After seven days into the remineralization process, specimens treated with artificial saliva primarily exhibited a porous structure with slight hints of the original enamel texture surrounding these areas (Figure 6A). Meanwhile, the application of flaxseed paste initiated the formation of dense, irregular globules across the remineralization zones, indicating active changes (Figure 6B). Chicken Eggshell Paste treatment preserved the enamel’s prismatic configuration, displaying a neat and systematic mineralization pattern with minimal breakdown areas, thus highlighting effective structural maintenance (Figure 6C). Similarly, the application of Fluoride Toothpaste resulted in a structured mineralization pattern, though some dissolution was observed within the enamel prisms (Figure 6D).

**Figure 7:**
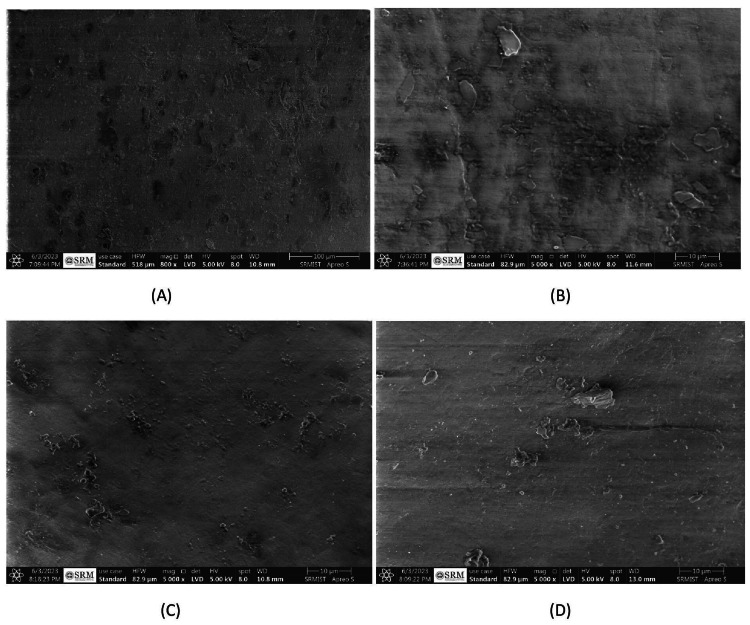
(A) SEM image of remineralized enamel after 14 days in GROUP 1, (B) SEM image of remineralized enamel after 14 days in GROUP 2, (C) SEM image of remineralized enamel after 14 days in GROUP 3, (D) SEM image of remineralized enamel after 14 days in GROUP 4.

Fourteen days into the remineralization process, the appearance of the samples showed varied progress across different treatments. Samples treated with artificial saliva continued to exhibit a porous appearance with minimal visibility of the original enamel structure (Figure 7A). The use of Flaxseed Paste led to a decrease in globule irregularity but still emphasized porosity within the remineralization areas (Figure 7B). Chicken Eggshell Paste resulted in diminutive, dispersed globules in an irregular pattern, with tiny remineralization spots noticeable (Figure 7C). Meanwhile, Fluoride Toothpaste treatment suggested a less organized remineralization pattern, with minor core restoration observed (Figure 7D).

**Figure 8:**
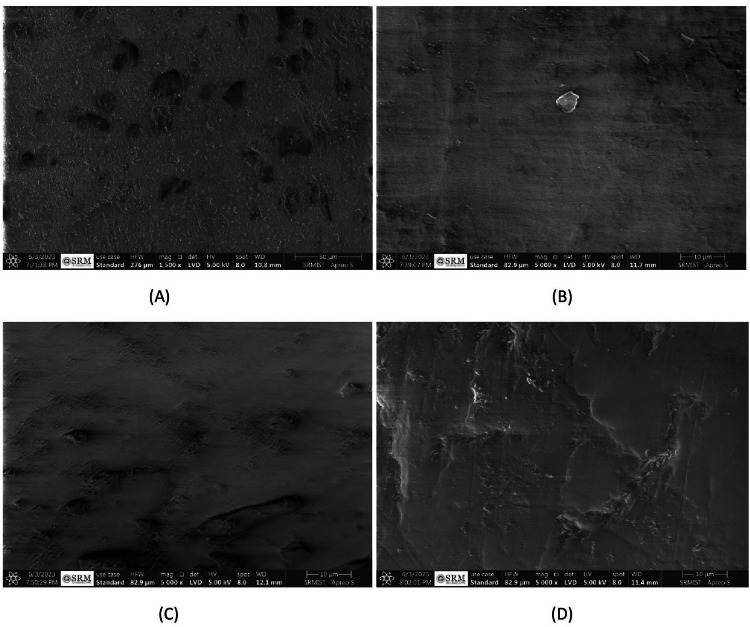
(A) SEM image of remineralized enamel after 28 days in GROUP 1, (B) SEM image of remineralized enamel after 28 days in GROUP 2, (C) SEM image of remineralized enamel after 28 days in GROUP 3, and (D) SEM image of remineralized enamel after 28 days in GROUP 4.

After 28 days into the remineralization process, noticeable changes were observed in the structural integrity and texture of enamel across different treatments. The porosity in samples soaked in artificial saliva was visibly reduced, indicating a decrease in demineralization effects (Figure 8A). Treatment with Flaxseed Paste yielded small, sporadic globules within the zones of remineralization (Figure 8B). Chicken Eggshell Paste consistently maintained an intact, well-aligned prismatic enamel structure, with negligible remineralization spots, presenting a deliberate mineralization approach (Figure 8C). Fluoride Toothpaste showed a patchy mineralization pattern with evident core remineralization activity (Figure 8D). These SEM findings highlight the distinct impacts of various remineralization treatments over time.

## Discussion

Dental caries stands out as a common chronic condition among children, primarily caused by an imbalance in the cycles of enamel demineralization and remineralization [1-3]. This issue not only negatively impacts the oral health of children but also their self-esteem. Strategies aimed at remineralization are focused on restoring the lost mineral content by reintroducing calcium and phosphate into the demineralized pores of enamel, whether through saliva or external sources. Fluorides have been widely recognized for their capacity to prevent demineralization and facilitate remineralization by replacing the hydroxide ions in enamel with fluoride ions [4-6]. This substitution leads to the creation of a fluorapatite crystal structure, characterized by its reduced solubility and enhanced resistance to acid-induced demineralization [6,7].

Despite its benefits, fluoride's effectiveness is inherently limited by the availability of phosphate and calcium ions and is notably more effective in addressing smooth surface caries than it is with pit and fissure caries [7]. These limitations associated with fluoride have driven the pursuit of alternative remineralization strategies that do not rely on fluoride [7]. In this context, the current in vitro investigation aimed to assess the potential of natural compounds in remineralizing caries lesions that have been experimentally induced in primary teeth [8,9]. Primary molar teeth were chosen for this study due to their soft, porous enamel and higher organic content compared to permanent teeth, which makes them more susceptible to caries [10].

For the experimental setup, specimens were subjected to a demineralization solution for 96 hours at 37°C, creating a subsurface demineralization that mimics early enamel lesions [10,11]. This setup was designed to replicate the natural occurrence of demineralization in the oral cavity, with a demineralization period set to three hours in the pH cycling phase [12,13].

The exploration of remineralization strategies in this study includes Casein Phosphopeptide-Amorphous Calcium Phosphate Complexes (CPP-ACP), tooth mousse, and various resin infiltration techniques, among others [12-14]. Recently, there has been an increasing interest in organic agents due to their bio-friendly properties, cost-effectiveness, availability, and broader safety margin [8,9]. Certain plant-derived polyphenols, found in natural agents like flaxseed and chicken eggshells, have been identified for their ability to regulate the demineralization-remineralization cycle of enamel [15,16]. Flaxseed extract, in particular, has shown promise in treating xerostomia and possesses antimicrobial effects against pathogens that cause dental decay [10]. Similarly, chicken eggshells have been highlighted for their oral health benefits, including antimicrobial and healing properties [17]. Nevertheless, the literature remains sparse regarding the remineralization potential of these organic ingredients [8,9].

This study adopts conventional in-vitro models for caries research, with artificial caries-like lesion production methodologies as initially described by Stookey et al. in 2011 [17]. The preparation of the demineralizing solution followed Stookey's pH mode, aiming to produce WSL of approximately 70 µm in depth, similar to those described by White et al. in 1987. The period for remineralization assessment was selected to evaluate repeated measures of outcomes, reflecting the dynamic nature of enamel remineralization [14].

The natural remineralization potential of saliva, as described by Cardogo et al., was compared with that of the treatments in this study. While remineralization was observed in the artificial saliva group, it was less pronounced than in the groups treated with flaxseed paste and CESP [18]. SEM-EDX evaluation using an HRSEM (manufactured by Thermoscientific Apreo S) did not show significant improvements in the weight percentage of calcium and phosphorus in the WSL, with SEM images revealing minimal traces of remineralization and increased porosities. This suggests that the mineral gain from artificial saliva might be superficial, pointing to the need for supplementary remineralizing agents to repair deeper layers of enamel [2,12].

The treatment groups receiving flaxseed paste and CESP demonstrated calcium and phosphate levels comparable to those observed with fluoride toothpaste after 28 days, with SEM images indicating more frequent areas of remineralization than the control group. This study bridges a gap in the literature by providing detailed insights into the surface mineral content and changes in surface morphology following treatment with these natural agents. Previous studies, such as those by Haghgoo et al., have explored the effects of flaxseed paste primarily based on colorimetric parameters, attributing its remineralization potential to its significant mineral content [19]. The change in ΔE of remineralized enamel at 28 days was found to be statistically significant compared to WSL, supporting the effectiveness of these natural remineralization agents [20-22].

Moreover, the study by Mony et al. in 2015 emphasized the crucial role of CESP in enamel remineralization, particularly when applied topically due to its high concentration of bioavailable calcium [14]. The calcination process of CESP aimed to produce a pure, pathogen-free powder while enhancing its alkalinity, conducive to increased ionic activity of remineralization-relevant anions. This alkaline environment is beneficial for enamel remineralization, especially in solutions with a lower pH, where the concentration of H+ ions is higher, facilitating the availability of ions necessary for enamel repair.

Scanning electron microscope (SEM) images of CESP revealed nanoparticles that were smooth and spherical, with sizes ranging between 100 nm and 200 nm [17]. These nanoparticles tended to cluster, forming aggregates that contributed to a cloudy appearance, attributed to the increased surface tension from the high temperature used in the decarbonization process. The high-temperature calcination also led to enhanced moisture absorption, resulting in the formation of the Ca(OH)2 phase with a hexagonal structure, indicative of particle agglomeration [16].

This finding aligns with the observations by Vestergaard et al. in 2008, who demonstrated that calcium carbonate nanoparticles adopted a spherical shape and tended to conglomerate, with a smooth surface and diameters within the same range. This consistency in nanoparticle characteristics underscores the reproducibility and relevance of using CESP in remineralization studies [21].

The use of various methodologies to study the remineralization of carious lesions, including microradiography, polarized light microscopy, microhardness tests, mineral analysis of calcium phosphate phases, and both transmission and scanning electron microscopy, highlights the complexity of enamel repair processes. Energy dispersive x-ray (EDX) spectroscopy has emerged as a recent technique for quantitatively assessing minor changes in mineral content, providing valuable insights into the elemental composition of treated enamel surfaces [21]. EDX spectroscopic analysis of CESP, flaxseed paste, and fluoride toothpaste facilitated a detailed evaluation of their remineralization efficacy. Here, the results indicate that the elemental composition of CESP closely matched the findings by Mony et al. in 2015, further validating the potential of these natural agents in dental care [18].

The application of remineralizing agents on enamel lesions led to the diffusion of mineral ions into the superficial layer, effectively reducing surface porosities and highlighting the significant differences in mean calcium/phosphate weight percentages between remineralized and demineralized enamel samples [15]. This outcome corroborates the findings from previous studies, demonstrating the effectiveness of CESP in promoting enamel surface remineralization [18].

Furthermore, the experiment noted significant changes in the calcium/phosphate weight percentages throughout the treatment period, with a marked decrease following exposure to the demineralizing solution and a substantial increase after the application of remineralizing agents [16]. This trend underscores the efficacy of both CESP and flaxseed paste in addressing incipient enamel caries lesions, suggesting their viability as alternative remineralization strategies to traditional fluoride-based treatments [17].

The lack of a significant difference in remineralization outcomes between the use of CESP solution and flaxseed paste, as confirmed by EDX and SEM analyses using an HRSEM (manufactured by Thermoscientific Apreo S), points to the limitations of further mineral diffusion once a certain level of surface repair has been achieved. This observation suggests that while these natural agents are effective in initiating enamel repair, their impact may plateau at a certain point, indicating the need for ongoing research to fully understand and optimize their remineralization potential.

This study contributes to the body of research assessing the protective effects of fluoride-containing products against initial enamel caries, reinforcing the notion that fluoride can offer a protective barrier against acid attacks but may not completely halt the demineralization process [4-6]. The findings advocate for a targeted approach to remineralization, aiming to shift from invasive dental procedures to more conservative, preventive measures. The rich bioavailability of calcium, along with the high concentration of phosphates in CESP, plays a crucial role in this process, offering a promising avenue for enamel repair and restoration.

The comparison of natural agents with sodium fluoride toothpaste, as conducted by Silva et al., reveals that while fluoride toothpaste may show an initial increase in calcium and phosphorus weight percentages, the long-term effects after 28 days are comparable to those achieved with flaxseed paste and chicken eggshell powder. SEM images further illustrate a uniform pattern of remineralization across treatments, with slight areas of dissolution, highlighting the potential of natural agents in achieving results similar to fluoride-based products [8].

The incorporation of chicken eggshell or flaxseed into mouthwash or toothpaste presents an opportunity to develop safe, effective, and economical dental care products. Future clinical trials could explore the full potential of these natural agents in preventing demineralization and promoting enamel repair, possibly through their inclusion in varnishes, primers, or composite resins. Such research could significantly advance our understanding of natural remineralization processes and lead to the development of innovative strategies for managing dental caries, particularly in pediatric dentistry. However, a limitation of this study is the inability to assess the number of remineralizing layers after the application of the remineralizing agents. While SEM-EDX provides detailed images and compositional analysis, it is inherently limited to evaluating surface phenomena and does not provide insight into deeper structural changes within the enamel and dentin. This surface-level analysis might not fully capture the entirety of the remineralization dynamics. Although every effort was made to simulate oral cavity conditions, certain variables inherent to in vitro studies, such as constant temperature and pH, do not perfectly mimic the natural fluctuations seen in human oral environments. These differences might affect the behavior of remineralization agents under real-world conditions.

Hence, the novelty of this in vitro investigation exhibits the potent remineralization capabilities of CESP, and flaxseed compared to traditional fluoride toothpaste in treating simulated carious lesions on primary teeth enamel. This study lays the groundwork for future research and clinical applications, potentially revolutionizing the approach to dental care by emphasizing natural, preventive, and restorative methods over conventional treatment modalities.

## Conclusions

In conclusion, our study highlights CESP and flaxseed paste as superior alternatives to fluoride toothpaste for the remineralization of primary enamel. These natural agents effectively not only enhance enamel restoration but also offer a safer, non-invasive option for treating carious lesions in pediatric dentistry. This evidence supports the potential of integrating CESP and flaxseed into preventive dental care strategies for children, promising a shift toward more holistic and effective treatments in pediatric dental health.
